# Vaccination Using Recombinants Influenza and Adenoviruses Encoding Amastigote Surface Protein-2 Are Highly Effective on Protection against *Trypanosoma cruzi* Infection

**DOI:** 10.1371/journal.pone.0061795

**Published:** 2013-04-24

**Authors:** Rafael Polidoro Alves Barbosa, Bruno Galvão Filho, Luara Isabela dos Santos, Policarpo Ademar Sales Junior, Pedro Elias Marques, Rafaela Vaz Sousa Pereira, Denise Carmona Cara, Oscar Bruña-Romero, Maurício Martins Rodrigues, Ricardo Tostes Gazzinelli, Alexandre Vieira Machado

**Affiliations:** 1 Departamento de Bioquímica e Imunologia, Instituto de Ciências Biológicas, Universidade Federal de Minas Gerais, Belo Horizonte, Minas Gerais, Brasil; 2 Departamento de Microbiologia, Instituto de Ciências Biológicas, Universidade Federal de Minas Gerais, Belo Horizonte, Minas Gerais, Brasil; 3 Departamento de Morfologia, Instituto de Ciências Biológicas, Universidade Federal de Minas Gerais, Belo Horizonte, Minas Gerais, Brasil; 4 Centro de Pesquisas René Rachou, FIOCRUZ, Belo Horizonte, Minas Gerais, Brasil; 5 Centro de Terapia Celular e Molecular (CTCMol), Universidade Federal de São Paulo, Escola Paulista de Medicina, São Paulo, Brasil; 6 Division of Infectious Diseases and Immunology, Department of Medicine, University of Massachusetts Medical School, Worcester, Massachusetts, United States of America; University of São Paulo, Brazil

## Abstract

In the present study we evaluated the protection raised by immunization with recombinant influenza viruses carrying sequences coding for polypeptides corresponding to medial and carboxi-terminal moieties of *Trypanosoma cruzi* ´s amastigote surface protein 2 (ASP2). Those viruses were used in sequential immunization with recombinant adenovirus (heterologous prime-boost immunization protocol) encoding the complete sequence of ASP2 (Ad-ASP2) in two mouse strains (C57BL/6 and C3H/He). The CD8 effector response elicited by this protocol was comparable to that observed in mice immunized twice with Ad-ASP2 and more robust than that observed in mice that were immunized once with Ad-ASP2. Whereas a single immunization with Ad-ASP2 sufficed to completely protect C57BL/6 mice, a higher survival rate was observed in C3H/He mice that were primed with recombinant influenza virus and boosted with Ad-ASP2 after being challenged with *T. cruzi*. Analyzing the phenotype of CD8+ T cells obtained from spleen of vaccinated C3H/He mice we observed that heterologous prime-boost immunization protocol elicited more CD8+ T cells specific for the immunodominant epitope as well as a higher number of CD8+ T cells producing TNF-α and IFN-γ and a higher mobilization of surface marker CD107a. Taken together, our results suggest that immunodominant subpopulations of CD8+ T elicited after immunization could be directly related to degree of protection achieved by different immunization protocols using different viral vectors. Overall, these results demonstrated the usefulness of recombinant influenza viruses in immunization protocols against Chagas Disease.

## Introduction

Over a hundred years after its first description, Chagas Disease remains as an important public health problem, mostly in Latin America. Nonetheless, the infection rate is increasing in other continents, mostly by blood transfusion [1], [2]. Accordingly to WHO, there are currently over 10 million people infected in Latin America and more than 100 million people live at risk areas in endemic countries. Moreover, this disease kills approximately 13 thousand people every year, due to the clinical complications and to the poor efficacy of the pharmacological treatment which is highly toxic and effective mostly during the acute phase of disease [3], [4]. In addition, the resistance of parasites to chemotherapy is another major drawback to the pharmacological treatment [5], [6], [7]. Thus, the development of vaccines is an important approach to be used in therapy and prophylaxis of Chagaś disease [3], [8].

Many vaccination studies against Chagas’ disease already provided evidence that CD8^+^ T cells play pivotal role on the development of protective immunity [9], [10], [11], [12]. Mechanisms used by these cells to eliminate the parasite include directly killing of infected cell or secretion of cytokines such as IFN-γ [13], [14]. Among the antigens that have been studied as potential candidates for vaccine development, the surface amastigote protein 2 (ASP2) has been found as one of the most promising [15], [16]. In addition, different strategies have already been tested to deliver this antigen in mice, including the use of recombinant protein, plasmid DNA and recombinant viruses [17], [18], [19], [20]. For instance, our group demonstrated that two sequential immunizations with recombinant HuA5 adenovirus encoding ASP2 were able to significantly reduce the parasitemia and improve the survival of vaccinated mice, when they were challenged with Y strain of *T. cruzi* [18]. However, in spite of these very promising results, a drawback in use the same viral vector in sequential immunizations rely on the risk that anti-vector antibodies generated after the priming could neutralize the vector when it is used in further immunizations and, consequently, hurdle the boost of heterospecific immune response [21], [22]. The limitation of anti-vector response elicited by homologous prime-boost immunization could be surpassed by different strategies, such as the use of two different recombinant viruses on prime and boost immunizations [23], [24].

Live recombinant influenza viruses have some features that make them attractive to be used in vaccination protocols against protozoan infections, as we can mention: They are well known inductors of Cytotoxic T Lymphocytes (CTLs) by direct infection of immature dendritic cells (DCs) and monocytes, facilitating antigen (Ag) presentation both local and systemically [25], [26], [27]; It is feasible to generate recombinant influenza viruses by reverse genetics techniques [28]; There are different influenza A strains and subtypes, which could be used in sequential immunizations to overcome previous immune responses directed to the vector [29].

Therefore, in the present study we exploited the use of recombinant influenza viruses carrying truncated sequences of ASP2 in sequential immunization with adenovirus encoding ASP2. This immunization protocol elicited potent anti-ASP2 cellular immune response, reduced the parasite burden and improved the survival of vaccinated mice when they were challenged with *T. cruzi*.

## Materials and Methods

### Mice and Ethics

Male of eight- to ten-weeks-old C57BL/6 and C3H/He mice were obtained from René Rachou Research Institute’s (CPqRR) animal facility center (Fiocruz, Belo Horizonte, Brazil) and housed according to institutional standard guidelines. All animal studies were approved by the Ethical Commission on Animals’ Use (CEUA) at Oswaldo Cruz Foundation (Fiocruz), license LW-9-09, and performed following institutional Guide for the Care and Use of Laboratory Animals.

### Cells and Parasites

MDCK and 293T cells (obtained from Pasteur Institut, FR) were grown at 37°C under 5% CO_2_ in complete Dulbeccós modified Eagle Medium (DMEM; SIGMA) with 1 mM sodium pyruvate, 4.5 mg/ml L-glucose, 100 U/ml penicillin and 100 µg/ml streptomycin (herein called complete DMEM medium) and respectively supplemented with 5% or 10% heat inactivated fetal calf serum (FCS; CULTILAB) [30]. Trypomastigotes from *T. cruzi* Y Strain were maintained as previously described [17] and challenge infections were performed by inoculating the mice with 1000 (C57BL/6) or 500 (C3H/He) bloodstream trypomastigotes by intraperitoneal route. Mice survival was monitored daily and parasite development was monitored by counting the number of bloodstream trypomastigotes in 5 µl of fresh blood collected from the tail vein [31].

### Plasmids for Influenza Reverse Genetics

**Figure 1:**
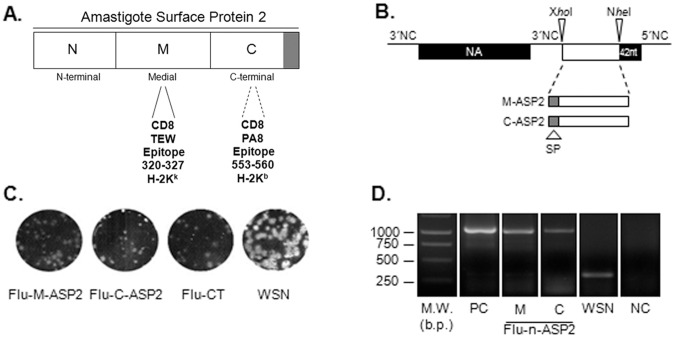
Construction and characterization of recombinant influenza viruses. Schematic representation of primary sequence of Amastigote Surface Protein 2 and its corresponding moieties, highlighting the mapped CD8 T cells epitopes (A). Schematic representation of the neuraminidase dicistronic segment. NA38 segment contains an A/WSN/33 (WSN) derived recombinant neuraminidase (NA) segment followed by a duplicated 3′non coding (NC) sequence, *Xho*I and *Nhe*I cloning sites, a duplication of the last 42 nucleotides of NA (dark box) and the original 5′NC sequence (28 nucleotides). The foreign sequences (open boxes) were cloned between *Xho*I and *Nhe*I cloning sites (B).The plaque phenotype of the wild type WSN and recombinant influenza viruses were assessed by standard agarose plaque assay in MDCK cells after 3 days of incubation at 35°C and 5% CO_2_ (C).The NA segments of recombinant influenza viruses were analyzed by RT-PCR, using a set of primers that allows the amplification of the region containing the inserted foreign sequence. Corresponding plasmids DNAs were amplified in parallel as positive control. The amplified products were analyzes on a 1% agarose and visualized by ethidium bromide staining. The values depicted at the weight marker lane ar (D). W.M: weight marker; M: medial moiety of ASP2, C: carboxi-terminal moiety of ASP2; b.p.: base pairs.

Wild type (pPRNA) and dicistronic (pPRNA38) plasmids from neuraminidase (NA) segments of A/WSN/33 virus (H1N1) were constructed as previously described [30], [32], [33]. Due the size constraints, we constructed plasmids encoding 660 nucleotides corresponding respectively to medial (M-ASP2) and carboxi-terminal (C-ASP2) segments of ASP2 (figure 1A). These sequences were obtained by PCR using the plasmid pAdCMV-ASP2 as template [18] and specific primers for each ASP2 portion. The amplicons were cloned into *Kpn*I and *Nhe*I digested pIgSP plasmid in frame to the sequence coding for κ chain of mice immunoglobulin that allows the secretion of the foreign sequence [17]. Those constructs were used as PCR templates to generate IgSP-M or C-ASP2 segments which were site directed cloned into *Xho*I and *Nhe*I digested pPRNA38 vector (Figure 1B). All primers sequences are available under request and the respective presenting haplotype were referenced within the correspondent portion (Figure 1A) [34], [35]. The generated plasmids (pPRNA38-M-ASP2 and pPRNA38-C-ASP2) were analyzed using Dynamic ET Dye Terminator Cycle Sequencing KIT® (AMERSHAM) and a Megabace 1000 automatic sequencer (AMERSHAM).

Influenza segments transfer plasmids pPOLI-HA, M, NS, PB2, PB1, PA and NP and the expression plasmids pcDNA-PA, NP, PB1 and PB2 were kindly provided by Dr George Brownlee (Sir William Dunn School of Pathology, University of Oxford, Oxford, United Kingdom) [36].

### Generation of Recombinant Viruses

Recombinant adenovirus harboring the entire ASP2 coding region (Ad-ASP2), recombinant adenovirus (Ad-CT) and influenza (Flu-CT) virus, which encode unrelated sequences were generated as previously described [18], [30], [37]. Recombinant influenza viruses carrying dicistronic NA38-ASP2 segments were generated by the twelve plasmid-driven genetic reverse technique, as described by Fodor and co-workers with modifications [30], [36]. Briefly, co-cultures of HEK 293T and MDCK cells were simultaneously transfected with plasmid coding the dicistronic NA segment (pPRNA38-M-ASP2 or pPRNA38-C-ASP2; 0.5 µg), the expression plasmids (pcDNA-PB1, pcDNA-PB2, pcDNA-NP and pcDNA-PA; 0.5 µg of each plasmid) and the other seven transfer plasmids of influenza A/WSN/33 segments (0.5 µg of each plasmid) using Fugene 6 Reagent® (ROCHE). Three days after incubation, infectious viral particles of recombinant vNA38-M-ASP2, vNA38-C-ASP2 (herein named respectively Flu-M-ASP2, Flu-C-ASP2) were recovered, amplified, plaque purified and titrated on MDCK as previously described [30].

### Viral RNA Extraction, RT-PCR Analysis

Viral RNA (vRNA) extraction from cell-free supernatants of infected MDCK cultures and RT-PCR analysis were performed as previously described [33]. Amplicons were analyzed on 1% agarose gel and visualized by ethidium bromide staining. RT-PCR products were purified and presence of mutations was determined by sequencing using Dynamic ET Dye Terminator Cycle Sequencing KIT® (AMERSHAM) and a Megabace 1000 automatic sequencer (AMERSHAM).

### Peptides

Peptides VNHRFTLV and TEWETGQI were purchased from Genscript (Piscataway, NJ). Peptide purity was in higher than 90%. Their identities were confirmed by Q-TOF Micro equipped with an electrospray ionization source (Micromass, United Kingdom).

### ELISPOT and Intracellular Cytokine Staining

ELISPOT assay was performed essentially as previously described [37]. Spleens cells of immunized mice were obtained three weeks after boost immunization. They were treated with ACK buffer for erythrocytes lysis and washed twice in RPMI containing 5% FBS before to be resuspended in cell culture medium consisting of RPMI 1640 medium (pH 7.4) supplemented with 10 mM HEPES, 0.2% sodium bicarbonate, 59 mg of penicillin/liter, 133 mg of streptomycin/liter, and 10% fetal bovine serum (CULTILAB) containing recombinant IL-2 (100 U/ml). The viability of the cells was evaluated by using 0.2% trypan blue exclusion dye to discriminate between live and dead cells. The number of spleen cells was adjusted to 1×10^6^ cells per well in cell culture medium and stimulated with specific peptides at final concentration of 10 µg/ml of VNHRFTLV (aa 553–560; for C57BL/6 splenocytes) or TEWETGQI (aa 320–327; for C3H/He splenocytes). The spots were counted on a S5 Core ELISPOT Analyser (CTL).

For Intracellular Cytokine Staining, the cell concentration was adjusted to 1×10^6^ cells per well in cell culture medium containing GolgiStop ™ and GolgiPlug™ (according to manufacturer instructions; BD Pharmingen) and -phycoerythrin (PE) anti-CD107a (BD Pharmigen). In half of the cultures, a final concentration of 10 µg/ml of VNHRFTLV (for C57BL/6 splenocytes) or TEWETGQI (for C3H/He splenocytes) peptide was added. The cells were cultivated in U-bottom 96-well plates (Corning) in a final volume of 200 µl at 37°C in a 5% CO_2_ humid atmosphere. After 12 hour-incubation, cells were stained for surface markers fluorescein isothiocyanate (FITC)-labeled dextramer TEWETGQI (Immudex), after 10 minutes incubation, cells were also stained with peridinin chlorophyll protein complex (PerCP) anti-CD8, avidin-phycoerythrin (PeCy7) anti-CD8, or FITC-labeled anti-CD3 (in samples without dextramer) antibodies (BD Pharmigen). The cells were fixed and permeabilized using Cytofix/Cytoperm kit (BD, Biosciences) according to manufacturer’s recommendations. Cells were then stained for intracellular markers allophycocyanin (APC) anti-IFN-γ, APC-Cy7 anti-TNF-α, or PE Cy7 anti-TNF-α (BD Pharmigen). Finally, the cells were fixed in 2% PBS-paraformaldehyde and at least 100,000 cells were acquired on a FacsCanto, LSRFortessa or FacsAria II (BD, Biosciences) flow cytometers and then analyzed with FlowJo software (ThreeStar). The ancestry gates are represented in Figure S1.

### ELISA and Western Blot

Recombinant ASP2 (rASP2) protein was produced in *Escherichia coli* as previously described [17]. The presence of sera specific anti-ASP2 antibodies were assessed by enzyme-linked immunosorbent assay (ELISA) on immunized mice sera obtained fourteen days after the boost immunization. Briefly, plates (Maxisorb, NUNC) were coated with 4 µg/mL (His65KDa, rASP2) and incubated at 4°C overnight. Mice sera were diluted 1∶100 in blocking buffer and incubated for 2 hours at 37°C. Plates were incubated with peroxidase-conjugated goat anti-mouse IgG (SIGMA) one hour at room temperature, and reactions were developed with 3,3′5,5′-tetrametylbenzidine (TMB) reagent (SIGMA) and read at 450 nm.

Alternatively, 0.5 µg of His65KDa, rASP2 were loaded on 12% polyacrylamide gels and transferred to nitrocellulose membranes. Membranes were then blocked and incubated with individual sera of mice immunized with recombinant viruses. After extensive washes, membranes were incubated with peroxidase-conjugated goat anti-mouse IgG (SIGMA) and detection was performed by membrane exposure to X-ray films after a standard chemoluminiscent reaction (ECL Detection System, Amersham Biosciences).

To measure IFN-γ production, spleen cells were obtained as described above and incubated for 72 hours at 37°C, 5% CO_2_. The IFN-γ concentration was determined in cell culture supernatant with DuoSet ELISA Development System mouse IFN-γ kit (R&D Systems) according to manufacturer’s recommendations.

### Immunizations

Heterologous prime-boost immunizations were performed as previously described [30]. Briefly, the animals were lightly anesthetized with a mixture of ketamine and xylazine and inoculated by intranasal route (IN) with 10^3^ plaque-forming unit (pfu) of recombinant influenza viruses (Flu-CT or Flu-nASP2) diluted in 25 µl of PBS. Four weeks later, the animals were boosted with 5×10^7^ pfu of recombinant Ad-ASP2 or Ad-CT in 100 µl of PBS by subcutaneous route (SC). Alternatively, some animals received two immunizations with 5×10^7^ pfu of recombinant Ad-ASP2 or AdCT by SC route four weeks apart (homologous prime-boost immunization protocol). Finally some mice received only one immunization with 5×10^7^ pfu of recombinant Ad-ASP2 by SC route.

### Statistical Analysis

Data are expressed as ± SEM and analyzed using GraphPad Prism ver.5 Software. Statistic significance for ELISA, ELISPOT and cytokine staining assays were evaluated using One-Way ANOVA and non-parametric test followed by Bonferroni post-test. Statistical significance for parasitemia was evaluated by 2-way ANOVA with Bonferroni post-test. The Gehan-Breslow-Wilcoxon test was performed to compare mouse survival curves.

## Results

### Generation and Characterization of Recombinant Influenza Viruses

Recombinant influenza viruses harboring the medial or the carboxi-terminal sequence of ASP-2 protein were recovered using the 12 plasmid driven reverse genetics as previously described [30]. These recombinant viruses, which were respectively named Flu-M-ASP2 and Flu-C-ASP2, displayed lysis plaques in MDCK cells similar in size than those found in cells infected with the recombinant Flu-CT. In contrast, those viruses displayed lysis plaques that were slightly smaller than those of the wild type WSN virus (Figure 1C). In addition, their infectious titers (1.4×10^6^ pfu/ml Flu-M-ASP2 and 2.8×10^6^ pfu/ml Flu-C-ASP2) were significantly lower than those of WSN virus(1×10^8^ pfu/ml).

As shown in figure 1D, amplifications products of expected size (∼1000 bp) were found for each recombinant influenza virus assayed. Moreover, when these amplicons were analyzed by sequencing, we found no mutations, demonstrating that those recombinant influenza viruses were genetically stable in cell culture (data not shown).

### Evaluation of Humoral Immune Response

**Figure 2:**
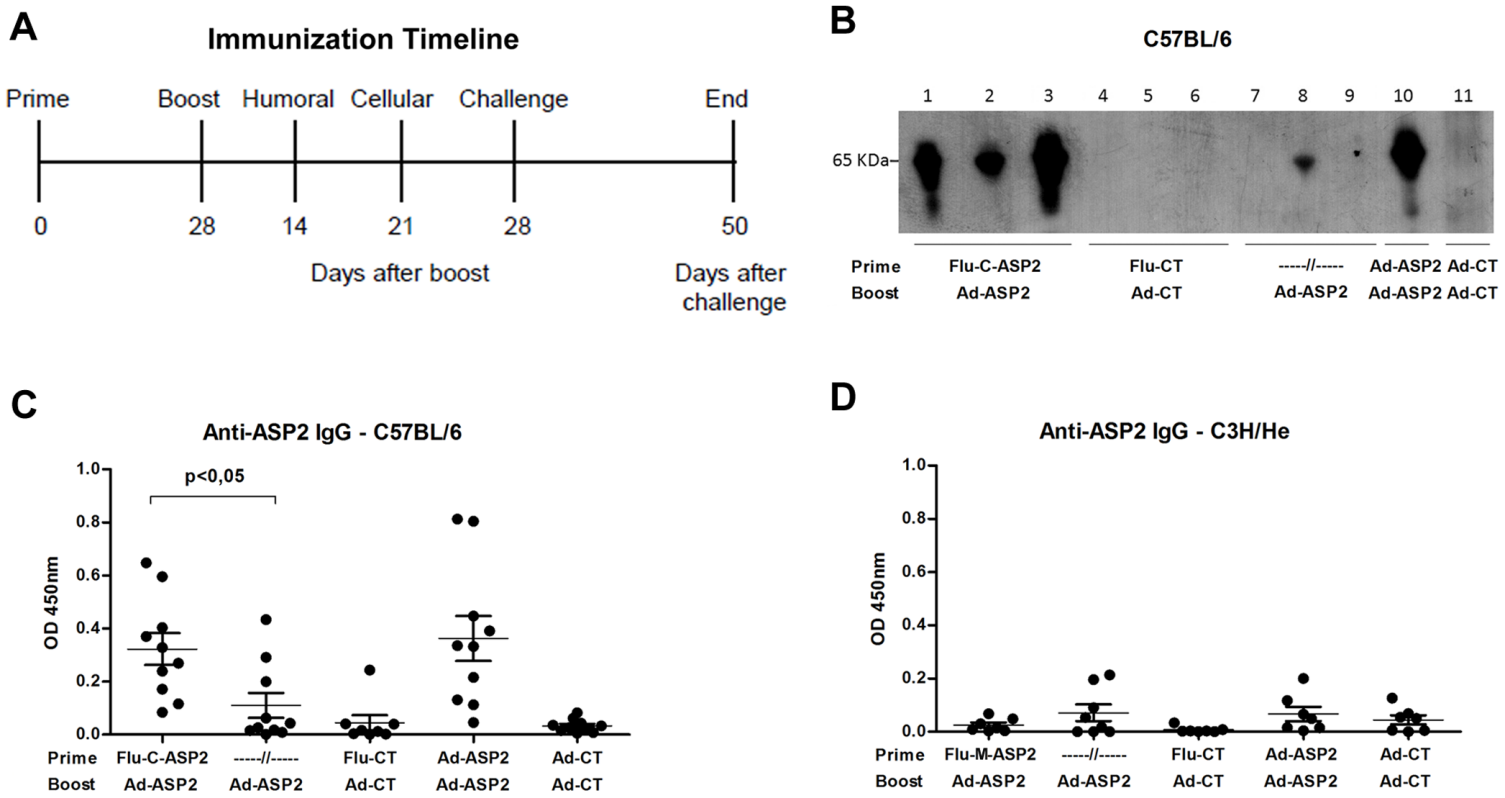
Immunization Schedule and Induction of specific anti-ASP2 humoral immune response in mice vaccinated with recombinant viruses. Timeline representation of immunization schedule and experimental procedures (A). C57BL/6 mice were immunized as described in Material and Methods. Two weeks after the boost immunization, the animals were bled and the presence of specific anti-ASP2 total IgG antibodies in mice sera was evaluated by western by incubating individual (lanes 1–9) or pooled (lanes 10 and 11) sera of C57BL/6 mice with nitrocellulose membranes loaded with recombinant ASP2 protein (His65KDa) as capture antigen blot (B). Alternatively, the antibodies levels were measured by ELISA using individual sera of C5BL/6 (C) or C3H/He (D) mice sera diluted 1∶100 and recombinant ASP2 protein as capture antigen. Optical Density (OD) was measured at 450 nm.

Immunization protocols were carried out according to the schedule depicted at figure 2A. Two weeks after the boost immunization, specific anti-ASP2 IgG serum antibodies were measured by ELISA and western blot, using the recombinant ASP2 (His65KDa) protein as capture antigen. Western blot results showed that specific anti-ASP2 IgG antibodies could be found in sera of all C57BL/6 mice primed with Flu-C-ASP2 and boosted with Ad-ASP2 (figure 2B), whereas only one animal that received a single immunization with Ad-ASP2 displayed detectable levels of specific anti-ASP2 antibodies. In addition, we detected higher levels of specific anti-ASP2 antibodies in the sera of mice primed with recombinant influenza than those found in animals that received only one immunization with Ad-ASP2 (Figure 2C). Interesting, neither by Western blot (data not shown) nor ELISA (figure 2D), we were able to detect specific anti-ASP2 antibodies in sera of C3H/He mice immunized with recombinant viruses, irrespective the immunization protocol used in vaccination. It is noteworthy that previous studies demonstrated that B epitopes are located in C-terminal moiety of ASP-2 protein and humoral immune response against intra-cellular amastigote proteins is not essential for protection [34], [38].

### Specific Cellular Immune Response Against Protective Epitopes

**Figure 3:**
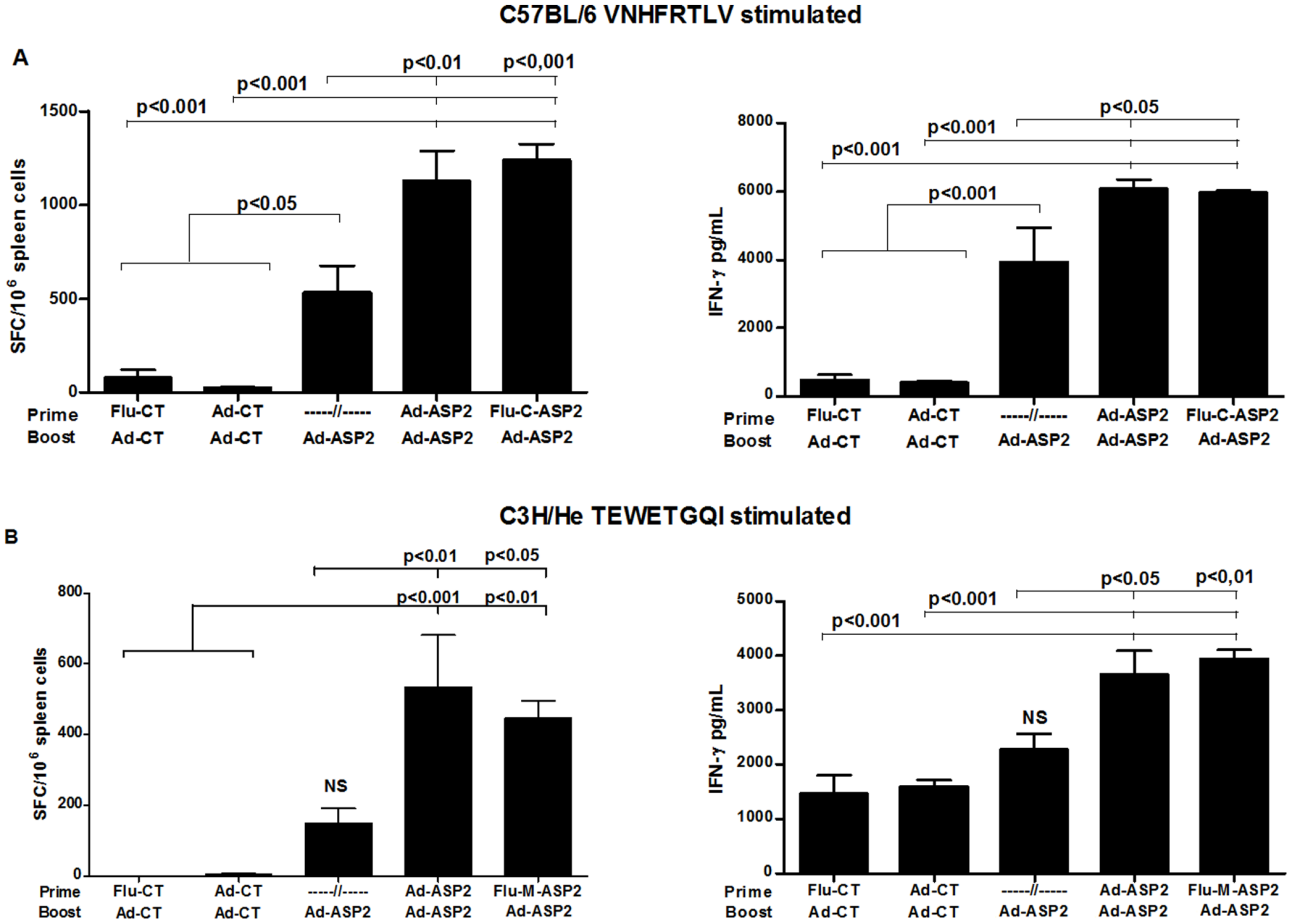
Cellular responses to immunodominant epitopes from ASP2 in mice immunized with recombinant viruses. C57BL/6 and C3H/He mice were immunized as described in Material and Methods. Three weeks after the boost immunization, the presence of ASP-2 specific IFN-γ producing T cells in spleen cells of C57BL/6 (A) or C3H/He (B) mice were assessed by ELISPOT and culture supernatant ELISA (n = 8). To this aim, the spleen cells of individual mice were stimulated 18 hours (ELISPOT) or 72 hours (ELISA) *ex vivo* with VNHRFTLV (aa 553–560; for C57BL/6) or TEWETGQI (aa 320–327; for C3H/He) specific ASP2 peptides. Optical Density (OD) was measured at 450 nm.

The activation of specific anti-ASP2 CD8+ T cell response was evaluated in spleen of immunized mice by stimulating their splenocytes with VNHRFTLV (H-2K^b^-restricted, C57BL/6) or TEWETGQI (H-2K^k^_-_restricted, C3H) peptides, three weeks after the boost immunization. As depicted in figure 3, specific IFN-γ producing CD8+ T cells could be found in spleen cells of mice primed with Flu-C-ASP2 or Flu-M-ASP2 and boosted with Ad-ASP2 (figure 3A and C). In addition, high amounts of IFN-γ could be measured in spleen cell culture supernatants stimulated *ex-vivo* with their respective peptides (Figure 3B and D). Interesting, in both cases, there was a clear improvement in the prime-boost immunization, as we could find a significant increase in IFN-γ production on prime-boosted groups compared to single Ad-ASP2 immunized mice (Figure 3).

### Protection Against Experimental Infection

**Figure 4:**
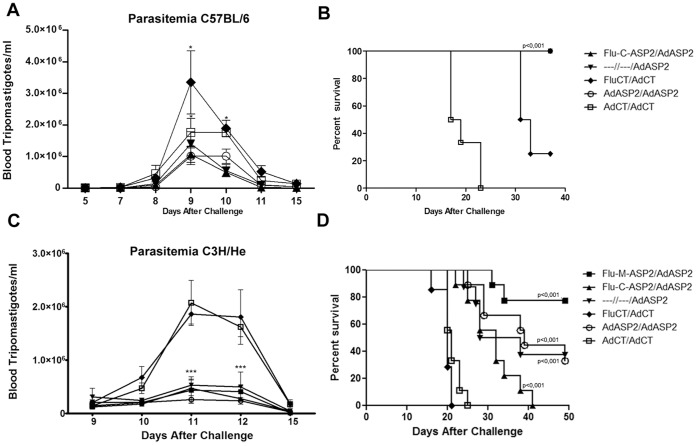
Parasitemia and survival curves of immunized mice challenged with *T.* *cruzi*. B6 and C3H/He mice were immunized as described in Material and Methods. Four weeks after boost immunization, they were challenged intraperitoneally with 1000 and 500, respectively, *T. cruzi* Y strain bloodstream trypomastigotes. Parasitemia was monitored on blood and depicted as the number of bloodstream trypomastigotes per milliliter of blood (A, n = 4; C, n = 8). The survival of vaccinated C57BL/6 (B, n = 7) and C3H/He (D, n = 7–9) mice was followed during 50 days and showed as Kaplan-Meier curves. * p<0.05, *** p<0.001.

The protection afforded by the vaccination protocols was evaluated by challenging the vaccinated mice with 500 (C3H/He) or 1000 (C57BL/6) bloodstream Y strain trypomastigotes. Regarding the resistant mice strain, C57BL/6, a single immunization with Ad-ASP2 sufficed to reduce the parasitemia and to completely protect the animals comparing to control immunized groups (Figure 4A, p<0.05; and 4B, p<0.001).

Regarding the C3H/He mice, which display remarkable susceptibility to *T. cruzi*, infection groups that received at least a single immunization with recombinant adenovirus-ASP2 were able to reduce the peak of parasitemia (Figure 4C, p<0.001), control tissue pathology (Figure S2) and prolong survival compared to the groups immunized with control recombinant viruses (Figure 4D p<0.0005). Remarkably, a higher survival rate was found in mice that were primed with Flu-M-ASP2 and boosted with Ad-ASP2 as close to 80% of vaccinated mice survived, comparing to mice that were primed with Flu-C-ASP2 and boosted with Ad-ASP2 (p = 0.0019). They also showed significant improvement of survival when compared to single or prime-boosted Ad-ASP2 immunized mice (p = 0.05, single and 0.08 homologous groups; Figure 4D).

In order to verify if the improvement of survival rate induced by the Flu-Ad protocol could be due to recombinant influenza properties, we tested the usefulness of a homologous intranasal prime subcutaneous-boost immunization using Flu-M-ASP2 virus in C3H/He mice strain. As demonstrated in Figure S3, we could not observe the production of specific immune response under stimulation neither by ELISPOT (Figure S3) nor intracellular staining for IFN-γ and TNF-α (data not shown) in splenocytes derived from homologous immunized mice. This could be expected since a single immunization with recombinant influenza is known to elicit neutralizing antibodies that can prevent a proper boost against the heterologous M-ASP2 polypeptide [30], [32].

### Cellular Immune Response Profile Elicited by Different Immunization Protocols

**Figure 5:**
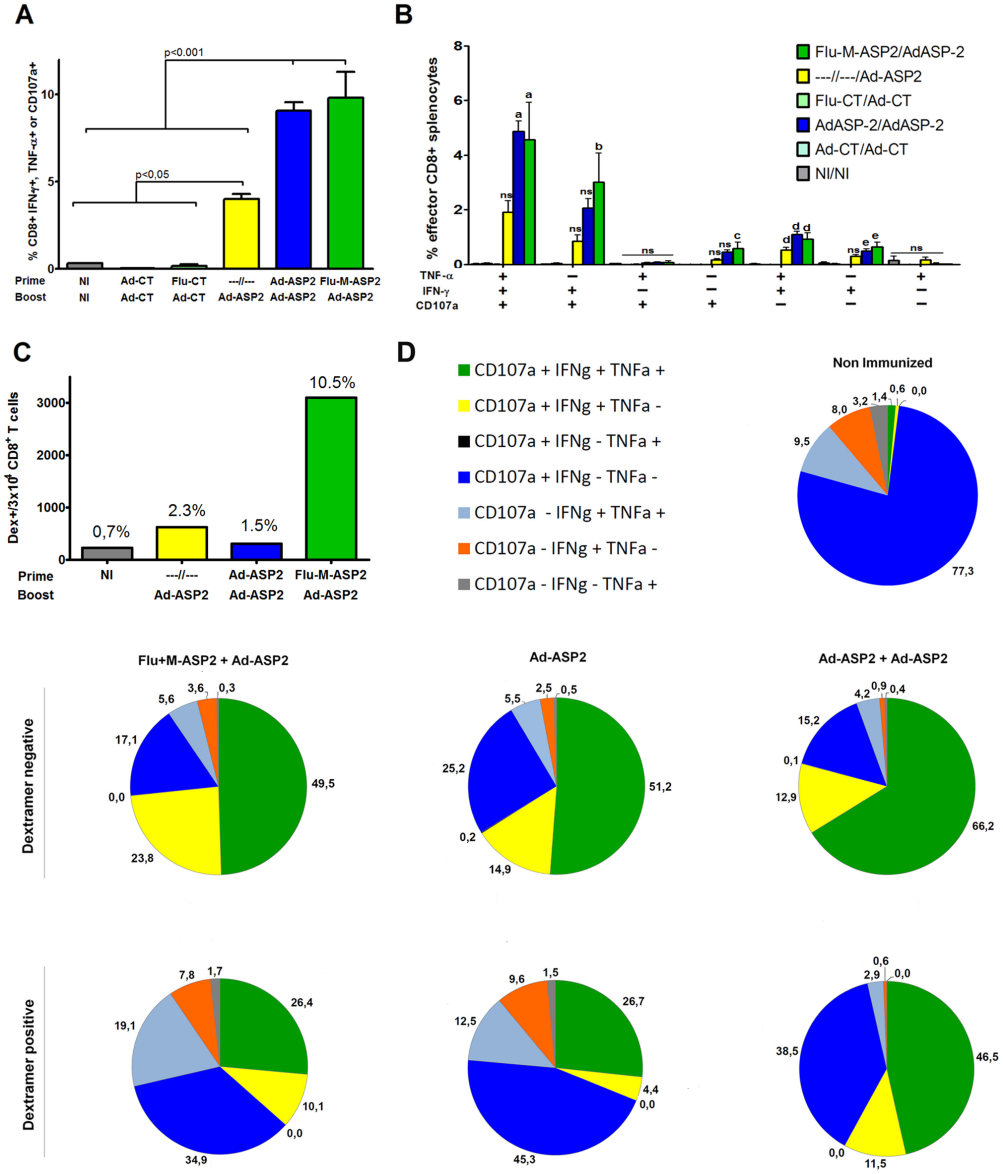
Phenotype of anti-ASP2 specific CD8+ T cells elicited by vaccination with recombinant viruses. C3H/He mice were immunized with recombinant viruses as described in Material and Methods. Two weeks after the last immunization, the spleen cells were harvested and cultivated *ex vivo* with specific TEWETGQI CD8+ T peptide and incubated with anti-CD3, anti-CD8, permeabilized and fixed and stained with anti-CD107a, anti-IFN-γ and anti-TNF-α antibodies and assessed by flow cytometry. Percentage of effector CD8+ T cells reacting to the presence of TEWETGQI peptide obtained from spleen cells of mice immunized with recombinant viruses (A). Percentage of CD8 T cells which produces IFN-γ or/and TNF-α or/and mobilizes the degranulation marker CD107a after stimulation with TEWETGQI (B), the statistics depicted are compared to groups of mice immunized with control recombinant viruses. The number and frequency of dextramer positive CD8+ T found in 3×10^4^ CD8+ T (C). Functional profile of CD8+ T cells subpopulations obtained from mice immunized with recombinant viruses (D). Response were depicted with different color patterns according to the number of assessed functions (IFN-γ, TNF-α and CD107a) displayed by each dextramer negative or dextramer positive CD8+ T cells subpopulations.

The survival results found in C3H/He mice prompted us to study more deeply the cellular immune profile elicited by the immunization protocols. To this aim, C3H/He mice were immunized as previously described and three weeks after the boost immunization, spleen CD8+ T cells were evaluated for intracellular staining of IFN-γ and TNF-α cytokines and for the surface mobilization of CD107a upon *ex vivo* stimulation with peptide TEWETGQI, as described in Material and Methods section. As depicted in figure 5A, the percentage of CD8+T cells positive for at least one of the parameters evaluated were similar in mice that received two immunizations with recombinant viruses, irrespective the immunization strategy employed.

Regarding the phenotype of subpopulations of CD8+ T cells found in vaccinated mice, triple (IFN-γ, TNF-α, CD107a) and double (IFN-γ+CD107a+, and IFN-γ+TNF-α) positive cells are the major populations that were found after immunization with recombinant viruses encoding ASP2 (Figure 5B). Interestingly, mice immunized with recombinant viruses encoding ASP2 displayed similar percentage of CD8+ T cell subpopulations, irrespective if they were immunized according to heterologous or homologous immunization protocols, and similar to IFN-γ production seen by ELISPOT and ELISA, there was a clear impact of boost immunization in the frequency of specific effector CD8+ T cells comparing prime-boosted groups with Ad-ASP2 single immunized group (Figure 5A and 5B).

In order to perform a more accurate analysis on CD8+ T cells elicited by immunization, we used a specific H-2K^d^/TEWETGQI dextramer. Mice were immunized as previously described and the phenotype of specific CD8+ T cells was assessed in TEWETGQI stimulated pooled spleen cells of vaccinated mice three weeks after the last immunization. As depicted in figure 5C, mice vaccinated with Flu-ASP2/Ad-ASP2 displayed the highest number of total dextramer positive CD8+ T cells. The main subpopulation of dextramer positive CD8+ T cells that were found in mice immunized irrespective the tested protocols were triple positives (IFN-γ, TNF-α, CD107), followed by single (CD107+) positives CD8+ T cells (Figure 5D). On the other hand, we could observe a higher frequency of single IFN-γ+TEWETGQI+ CD8+ T cells (CD107a- TNF-α-) in heterologous (19%) and Ad-ASP2 single (12.5%) immunized groups compared to Ad-ASP2/Ad-ASP2 group (2,9%).

Accordingly, Table 1 shows that besides heterologous Flu-Ad immunization elicited higher numbers of CD8+TEWETGQI+ T cells, also the frequency of CD8+TEWETGQI+ CD107a and/or IFN-γ and/or TNF-α positive cells increase above two fold compared to homologous or single immunized groups. We could also find a significant increase of perforin production under stimulation only in the heterologous vaccinated group (Figure S4). This results suggest the importance of those effector factors on protection and could also indicate that the improvement of survival by heterologous could be due to a higher number of effector specific CD8+ T cells.

**Table 1:** Percentage of effector CD8+ T cells in splenocytes of immunized mice.

% of effector CD8+ T cells in mice immunized with recombinant viruses								
	%Total	% CD8+ T cell Dex. Neg.			%Total	% CD8+ T cell Dex. Pos.		
Immun. Protocol	CD8+ T cell Dex. Neg.	CD107+	IFNγ	TNFα	CD8+ T cell Dex. Pos.	CD107+	IFNγ	TNFα
Flu-ASP2+ Ad-ASP2	89.5	9.9	9.0	6.1	10.5	2.1	1.8	1.3
-----+Ad-ASP2	97.7	5.1	4.1	3.2	2.3	0.5	0.4	0.3
Ad-ASP2+ Ad-ASP2	98.5	11.5	10.3	8.7	1.5	0.6	0.4	0.3
Non infected	99.3	1.1	0.3	0.2	0.7	0.2	0.0	0.0

## Discussion

Recombinant viruses carrying foreign sequences have been proven to be useful tools as vaccines against many pathogens, including those which require the induction of potent type I T cell immune responses, such as *Leishmania s.p.*, *Toxoplasma gondii* and *Trypanosoma cruzi* [39]. Studies carried out by our group demonstrated that two immunizations with recombinant adenovirus carrying *T. gondii* or *T. cruzi* antigens were able to elicit specific humoral and cellular immune response and to protect different mouse lineages after challenge with those protozoan parasites [18], [37]. In spite of these very promising results, two immunizations with recombinant adenovirus (homologous immunization protocol) raises some concerns, mostly due to the elicited anti-vector immune response, which could hurdle the immune response directed against the foreign sequence in subsequent vaccinations. This problem could be surpassed by using two different vectors for each immunization [24]. Therefore, we evaluated the use of recombinant influenza viruses encoding ASP2 derived polypeptides as a tool for priming the specific anti-ASP2 immune response, followed by sequential immunization with a recombinant adenovirus encoding ASP2. Regarding the naturally resistant C57BL/6 mice, the prime with recombinant influenza virus encoding the carboxi-terminal portion of ASP2 was as useful as recombinant adenovirus in priming specific anti-ASP2 immune response. Indeed, antibodies levels and the number IFN-γ producers CD8+ T cells specific for ASP2 were similar in mice primed with recombinant influenza or adenovirus. Interesting, regarding C57BL/6 mice strain, a single immunization with recombinant adenovirus suffice to control parasitemia and to completely protect the animals after challenge. Similar findings were obtained by Duan and collaborators using recombinant Sendai virus encoding ASP2, which was able to significantly reduce the parasitemia and to completely protect C57BL/6 mice after the challenge with Tulahuen strain [19].

Regarding the susceptible strain C3H/He, all immunizations protocols employing Ad-ASP2 in our study were able to significantly reduce the parasitemia, control at certain extent tissue pathology and prolong survival of challenged animals. Considering Y strain of *Trypanosoma cruzi*, there is a variable correlation between blood parasitemia and survival rate, as demonstrated in different mice strains [40], [41]. However, we could observe a correlation of parasitemia control with prolonged survival in our model. Notwithstanding, an improvement on the survival rate was observed in mice primed with recombinant influenza-M-ASP2 and boosted with recombinant adenovirus even when compared to the survival of the animals that were immunized once or twice with recombinant adenovirus, or primed using Flu-C-ASP2 which does not contain an immunodominant epitope to C3H/He MHC-I haplotype. These results seemed quite surprising because the specific anti-ASP2 cellular immune response, measured by the number of specific CD8+ T as well as by production of IFN-γ was similar in mice that were submitted either to the homologous or heterologous prime and boost immunization protocols.

Phenotype analyses performed on total CD8+ T cells obtained from vaccinated C3H/He showed that most effector CD8+ T cells were polyfunctional and mostly triple (IFN-γ, TNF-α and CD107a) and double (IFN-γ, CD107a) positives. These results were similar to those obtained previously in C57BL/6 mice that were immunized with naked DNA and adenovirus encoding ASP2 [42]. Our results also showed that mice that received one immunization with Ad-ASP2 displayed similar CD8+ T cells phenotype than those observed in mice that received two immunizations with recombinant viruses encoding ASP2, suggesting that just one immunization with Ad-ASP2 suffice for shaping the CD8+ T cells phenotype. Thus, our observations indicate that a single immunization using Ad-ASP2 suffice to stimulate a significant production of effector cytokines IFN-γ, TNF-α and mobilize CD107a, elicit an immunodominant effector population which can control parasitemia, reduce tissue pathology and prolong survival when compared to control immunized mice even in a susceptible model. This is particularly important because often, studies using different recombinant viruses or other vaccine vectors, mice models and *Trypanosoma cruzi* strains without the single immunized group could be overestimating their protection using prime-boost protocols.

Remarkably, C3H/He mice that were primed with Flu-M-ASP2 displayed higher number of dextramer positives CD8+ T cells than mice that were immunized with Ad-ASP2. Moreover, our results showed that a boost immunization with Ad-ASP2 did not augment the number of TEWETGQI dextramer positive CD8+ T cells in mice primed with Ad-ASP2. To discuss the reason by which the heterologous prime-boost protocol could improve protection and enhance the frequency of TEWETGQI CD8+ T cells we hypothesized that immunization with Flu-M-ASP2, which encodes only the medial moiety of ASP2, primed the immunodominant CD8+ response towards TEWETGQI epitope resulting in the expansion of this population after boosting with Ad-ASP2. In contrast, priming with Ad-ASP2, which carries the entire sequence of ASP2, could possibly elicit immune response also against subdominant epitopes of ASP2, resulting in a lower secondary response against TEWETGQI immunodominant epitope after boost [34], [38], [42], [43], [44]. Accordingly, previous results of our group suggest that immunization with plasmids or adenovirus encoding ASP2 subdominants epitopes afforded lower degree of protection when compared to that observed in animals immunized with vectors encoding the immunodominant epitope [42]. A reinforcement to this hypothesis could be found in the low number of proteins encoded by influenza when compared to adenovirus, which potentially reduces the number of viral antigens that could compete with the heterologous antigen for presentation by antigen-presenting cells [45], [46]. The correlation between TEWETGQI (present in medial portion of ASP2, M-ASP2) immunodominant frequency and protection is reinforced by the result of C3H/He mice that were immunized using recombinant influenza encoding the C-terminus portion of ASP2 as prime and Ad-ASP2 as boost presented a survival curve similar to single immunized or homologous prime-boost using Ad-ASP2 after infection (p = 0.46).

Another finding of our study was that the number of dextramer stained CD8+ T cells producing IFN-γ, TNF-α or the surface marker CD107a found in animals primed with Flu-M-ASP2 were approximately three times higher than those observed in other vaccinated groups. However, while the role of different T cell subpopulation to control the infection with some viruses, bacteria and Plasmodium was already well documented [47], [48], [49], [50], the biological relevance of CD8+ T cells subpopulations phenotypes to control the infection with *T. cruzi* remains elusive [51]. The IFN-γ production itself is known to be important for protection against *Trypanosoma cruzi* infection in many previous work of our and other groups [14], [52], [53], [54], [55]. On the other hand, other factors as the effector phenotype of specific CD8+ T cells, the production of perforin, the re-circulation of those cells out of spleen [56], their presence in the heart [57], [58], apoptosis of specific immunodominant anti-ASP2 CD8+ T cells [59], and the type of memory cells involved are important to be considered [51]. Recently a group has elegantly shown that multiple redundant effector CD8+ T cells factors deriving from transferred Tc1 and Tc17 populations are capable of protecting mice against viral infection [60], and as CD8+ T cells have a major role in protection against *Trypanosoma cruzi* infection, this statement is an interesting subject of research. Thus, if the improvement of protection observed in mice primed with recombinant influenza-M-ASP2 virus was only due to the higher number of CD8+T specific for the immunodominant epitope or could also be due to other factors remains to be solved.

In summary, we demonstrated that recombinant influenza viruses encoding an ASP2 derived polypeptide would be useful in heterologous prime-boost studies aiming the development of vaccines against Chagas Disease. The priming with recombinant influenza virus followed by boost with recombinant adenovirus could properly augment the number of effector CD8+ T cells specific for ASP2 immunodominant epitope, whose displayed unique phenotype and resulted in increased survival of vaccinated C3H/He mice challenged with *T. cruzi*.

## Supporting Information

Figure S1 **Representative of ancestry gates for flow cytometry experiments.** Correspondent ancestry gates for the figure 5 analysis.
(TIF)

Figure S2 **Histopathological analyses of liver, spleen and heart derived from infected mice.** Male C3H/He mice were primed and boosted according different immunization protocols and infected with 500 bloodstream trypomastigotes of Y strain of *T. cruzi*. Fifteen days after the infection, mice were euthanized and spleen, liver and heart were harvested, fixed and processed for histopathology. The organ sections were stained using hematoxilin-eosin and the degree of tissue inflammation was evaluated (scale bar - 100 µm).
(TIF)

Figure S3 **Cellular responses to immunodominant epitope from ASP2 in mice immunized twice using Flu-M-ASP2.** ELISPOT of stimulated splenocytes taken from C3H/He mice immunized with the depicted protocols. The prime-boost was performed within an interval of 28 days and the experiment was performed 21 days post boost. The splenocytes were incubated 18 h in the presence of 10 µg of TEWETGQI peptide (n = 5 for all groups except non-immunized group NI/NI, n = 3).
(TIF)

Figure S4 **Perforin production in splenocytes derived from C3H/He immunized mice.** Splenocytes derived from immunized C3H/He mice were *ex vivo* stimulated or not in the presence of Brefeldin A and Monesin A and the immunodominant peptide TEWETGQI for 12 hours, prepared, labeled and submitted to flow cytometry (n = 4). N.I. Non-immunized/Non-infected. Their staining profiles were analyzed using FlowJo and statistical analysis performed was 2-Way ANOVA with Bonferroni post-test using GraphPad Prism 5.0 Software.
(TIF)

## References

[1] Gascon J, Bern C, Pinazo MJ (2012) Chagas disease in Spain, the United States and other non-endemic countries. Acta Trop 115: 22–27. 10.1016/j.actatropica.2009.07.019 19646412

[2] Bern C, Kjos S, Yabsley MJ, Montgomery SP (2011) Trypanosoma cruzi and Chagas’ Disease in the United States. Clin Microbiol Rev 24: 655–681. 21976603 10.1128/CMR.00005-11 PMC3194829

[3] Vazquez-Chagoyan JC, Gupta S, Garg NJ (2011) Vaccine development against Trypanosoma cruzi and Chagas disease. Adv Parasitol 75: 121–146. 21820554 10.1016/B978-0-12-385863-4.00006-X

[4] Rassi A Jr, Rassi A, Marin-Neto JA (2010) Chagas disease. Lancet 375: 1388–1402. 20399979 10.1016/S0140-6736(10)60061-X

[5] Camandaroba EL, Reis EA, Goncalves MS, Reis MG, Andrade SG (2003) Trypanosoma cruzi: susceptibility to chemotherapy with benznidazole of clones isolated from the highly resistant Colombian strain. Rev Soc Bras Med Trop 36: 201–209. 12806455 10.1590/s0037-86822003000200002

[6] Le Loup G, Pialoux G, Lescure FX (2011) Update in treatment of Chagas disease. Curr Opin Infect Dis 24: 428–434. 21857512 10.1097/QCO.0b013e32834a667f

[7] Urbina JA (2001) Specific treatment of Chagas disease: current status and new developments. Curr Opin Infect Dis 14: 733–741. 11964893 10.1097/00001432-200112000-00012

[8] Bethony JM, Cole RN, Guo X, Kamhawi S, Lightowlers MW, et al (2011) Vaccines to combat the neglected tropical diseases. Immunol Rev 239: 237–270. 21198676 10.1111/j.1600-065X.2010.00976.x PMC3438653

[9] Rodrigues MM, Boscardin SB, Vasconcelos JR, Hiyane MI, Salay G, et al (2003) Importance of CD8 T cell-mediated immune response during intracellular parasitic infections and its implications for the development of effective vaccines. An Acad Bras Cienc 75: 443–468. 14605680 10.1590/s0001-37652003000400005

[10] Parodi C, Padilla AM, Basombrio MA (2009) Protective immunity against Trypanosoma cruzi. Mem Inst Oswaldo Cruz 104 Suppl 1288–294. 19753487 10.1590/s0074-02762009000900038

[11] Miyahira Y (2008) Trypanosoma cruzi infection from the view of CD8+ T cell immunity–an infection model for developing T cell vaccine. Parasitol Int 57: 38–48. 17728174 10.1016/j.parint.2007.07.005

[12] Junqueira C, Caetano B, Bartholomeu DC, Melo MB, Ropert C, et al (2010) The endless race between Trypanosoma cruzi and host immunity: lessons for and beyond Chagas disease. Expert Rev Mol Med 12: e29. 20840799 10.1017/S1462399410001560

[13] Muller U, Sobek V, Balkow S, Holscher C, Mullbacher A, et al (2003) Concerted action of perforin and granzymes is critical for the elimination of Trypanosoma cruzi from mouse tissues, but prevention of early host death is in addition dependent on the FasL/Fas pathway. Eur J Immunol 33: 70–78. 12594834 10.1002/immu.200390009

[14] de Alencar BC, Persechini PM, Haolla FA, de Oliveira G, Silverio JC, et al (2009) Perforin and gamma interferon expression are required for CD4+ and CD8+ T-cell-dependent protective immunity against a human parasite, Trypanosoma cruzi, elicited by heterologous plasmid DNA prime-recombinant adenovirus 5 boost vaccination. Infect Immun 77: 4383–4395. 19651871 10.1128/IAI.01459-08 PMC2747960

[15] Wizel B, Palmieri M, Mendoza C, Arana B, Sidney J, et al (1998) Human infection with Trypanosoma cruzi induces parasite antigen-specific cytotoxic T lymphocyte responses. J Clin Invest 102: 1062–1071. 9727076 10.1172/JCI3835 PMC508973

[16] Garg N, Tarleton RL (2002) Genetic immunization elicits antigen-specific protective immune responses and decreases disease severity in Trypanosoma cruzi infection. Infect Immun 70: 5547–5555. 12228281 10.1128/IAI.70.10.5547-5555.2002 PMC128309

[17] Boscardin SB, Kinoshita SS, Fujimura AE, Rodrigues MM (2003) Immunization with cDNA expressed by amastigotes of Trypanosoma cruzi elicits protective immune response against experimental infection. Infect Immun 71: 2744–2757. 12704149 10.1128/IAI.71.5.2744-2757.2003 PMC153249

[18] Machado AV, Cardoso JE, Claser C, Rodrigues MM, Gazzinelli RT, et al (2006) Long-term protective immunity induced against Trypanosoma cruzi infection after vaccination with recombinant adenoviruses encoding amastigote surface protein-2 and trans-sialidase. Hum Gene Ther 17: 898–908. 16972758 10.1089/hum.2006.17.898

[19] Duan X, Yonemitsu Y, Chou B, Yoshida K, Tanaka S, et al (2009) Efficient protective immunity against Trypanosoma cruzi infection after nasal vaccination with recombinant Sendai virus vector expressing amastigote surface protein-2. Vaccine 27: 6154–6159. 19712768 10.1016/j.vaccine.2009.08.026

[20] Nogueira RT, Nogueira AR, Pereira MC, Rodrigues MM, Galler R, et al (2011) Biological and immunological characterization of recombinant Yellow Fever 17D viruses expressing a Trypanosoma cruzi Amastigote Surface Protein-2 CD8+ T cell epitope at two distinct regions of the genome. Virol J 8: 127. 21418577 10.1186/1743-422X-8-127 PMC3066119

[21] Schneider J, Gilbert SC, Hannan CM, Degano P, Prieur E, et al (1999) Induction of CD8+ T cells using heterologous prime-boost immunisation strategies. Immunol Rev 170: 29–38. 10566139 10.1111/j.1600-065x.1999.tb01326.x

[22] Ramshaw IA, Ramsay AJ (2000) The prime-boost strategy: exciting prospects for improved vaccination. Immunol Today 21: 163–165. 10740236 10.1016/s0167-5699(00)01612-1

[23] Lu S (2009) Heterologous prime-boost vaccination. Curr Opin Immunol 21: 346–351. 19500964 10.1016/j.coi.2009.05.016 PMC3743086

[24] Radosevic K, Rodriguez A, Lemckert A, Goudsmit J (2009) Heterologous prime-boost vaccinations for poverty-related diseases: advantages and future prospects. Expert Rev Vaccines 8: 577–592. 19397415 10.1586/erv.09.14

[25] Manicassamy B, Manicassamy S, Belicha-Villanueva A, Pisanelli G, Pulendran B, et al (2010) Analysis of in vivo dynamics of influenza virus infection in mice using a GFP reporter virus. Proc Natl Acad Sci U S A 107: 11531–11536. 20534532 10.1073/pnas.0914994107 PMC2895123

[26] Kreijtz JH, Fouchier RA, Rimmelzwaan GF (2011) Immune responses to influenza virus infection. Virus Res 162: 19–30. 21963677 10.1016/j.virusres.2011.09.022

[27] Johnson S, Zhan Y, Sutherland RM, Mount AM, Bedoui S, et al (2009) Selected Toll-like receptor ligands and viruses promote helper-independent cytotoxic T cell priming by upregulating CD40L on dendritic cells. Immunity 30: 218–227. 19200758 10.1016/j.immuni.2008.11.015 PMC2694753

[28] Horimoto T, Kawaoka Y (2009) Designing vaccines for pandemic influenza. Curr Top Microbiol Immunol 333: 165–176. 19768405 10.1007/978-3-540-92165-3_8 PMC6133292

[29] Ferko B, Stasakova J, Sereinig S, Romanova J, Katinger D, et al (2001) Hyperattenuated recombinant influenza A virus nonstructural-protein-encoding vectors induce human immunodeficiency virus type 1 Nef-specific systemic and mucosal immune responses in mice. J Virol 75: 8899–8908. 11533153 10.1128/JVI.75.19.8899-8908.2001 PMC114458

[30] Machado AV, Caetano BC, Barbosa RP, Salgado AP, Rabelo RH, et al (2010) Prime and boost immunization with influenza and adenovirus encoding the Toxoplasma gondii surface antigen 2 (SAG2) induces strong protective immunity. Vaccine 28: 3247–3256. 20189485 10.1016/j.vaccine.2010.02.003

[31] Krettli AU, Brener Z (1976) Protective effects of specific antibodies in Trypanosoma cruzi infections. J Immunol 116: 755–760. 815433

[32] Vieira Machado A, Naffakh N, Gerbaud S, van der Werf S, Escriou N (2006) Recombinant influenza A viruses harboring optimized dicistronic NA segment with an extended native 5′ terminal sequence: induction of heterospecific B and T cell responses in mice. Virology 345: 73–87. 16271378 10.1016/j.virol.2005.09.050

[33] Machado AV, Naffakh N, van der Werf S, Escriou N (2003) Expression of a foreign gene by stable recombinant influenza viruses harboring a dicistronic genomic segment with an internal promoter. Virology 313: 235–249. 12951036 10.1016/s0042-6822(03)00289-7

[34] Araujo AF, de Alencar BC, Vasconcelos JR, Hiyane MI, Marinho CR, et al (2005) CD8+-T-cell-dependent control of Trypanosoma cruzi infection in a highly susceptible mouse strain after immunization with recombinant proteins based on amastigote surface protein 2. Infect Immun 73: 6017–6025. 16113322 10.1128/IAI.73.9.6017-6025.2005 PMC1231112

[35] Low HP, Santos MA, Wizel B, Tarleton RL (1998) Amastigote surface proteins of Trypanosoma cruzi are targets for CD8+ CTL. J Immunol 160: 1817–1823. 9469442

[36] Fodor E, Devenish L, Engelhardt OG, Palese P, Brownlee GG, et al (1999) Rescue of influenza A virus from recombinant DNA. J Virol 73: 9679–9682. 10516084 10.1128/jvi.73.11.9679-9682.1999 PMC113010

[37] Caetano BC, Bruna-Romero O, Fux B, Mendes EA, Penido ML, et al (2006) Vaccination with replication-deficient recombinant adenoviruses encoding the main surface antigens of toxoplasma gondii induces immune response and protection against infection in mice. Hum Gene Ther 17: 415–426. 16610929 10.1089/hum.2006.17.415

[38] Tzelepis F, de Alencar BC, Penido ML, Gazzinelli RT, Persechini PM, et al (2006) Distinct kinetics of effector CD8+ cytotoxic T cells after infection with Trypanosoma cruzi in naive or vaccinated mice. Infect Immun 74: 2477–2481. 16552083 10.1128/IAI.74.4.2477-2481.2006 PMC1418894

[39] Liniger M, Zuniga A, Naim HY (2007) Use of viral vectors for the development of vaccines. Expert Rev Vaccines 6: 255–266. 17408374 10.1586/14760584.6.2.255

[40] Roffe E, Rothfuchs AG, Santiago HC, Marino AP, Ribeiro-Gomes FL, et al (2012) IL-10 limits parasite burden and protects against fatal myocarditis in a mouse model of Trypanosoma cruzi infection. J Immunol 188: 649–660. 22156594 10.4049/jimmunol.1003845 PMC3253255

[41] Goncalves da Costa SC, Calabrese KS, Zaverucha do Valle T, Lagrange PH (2002) Trypanosoma cruzi: infection patterns in intact and athymic mice of susceptible and resistant genotypes. Histol Histopathol 17: 837–844. 12168794 10.14670/HH-17.837

[42] Dominguez MR, Silveira EL, de VasconcelosJR, de Alencar BC, Machado AV, et al (2011) Subdominant/cryptic CD8 T cell epitopes contribute to resistance against experimental infection with a human protozoan parasite. PLoS One 6: e22011. 21779365 10.1371/journal.pone.0022011 PMC3136500

[43] Rosenberg CS, Martin DL, Tarleton RL (2010) CD8+ T cells specific for immunodominant trans-sialidase epitopes contribute to control of Trypanosoma cruzi infection but are not required for resistance. J Immunol 185: 560–568. 20530265 10.4049/jimmunol.1000432 PMC3784248

[44] Schirmbeck R, Reimann J, Kochanek S, Kreppel F (2008) The immunogenicity of adenovirus vectors limits the multispecificity of CD8 T-cell responses to vector-encoded transgenic antigens. Mol Ther 16: 1609–1616. 18612271 10.1038/mt.2008.141

[45] Kastenmuller W, Gasteiger G, Gronau JH, Baier R, Ljapoci R, et al (2007) Cross-competition of CD8+ T cells shapes the immunodominance hierarchy during boost vaccination. J Exp Med 204: 2187–2198. 17709425 10.1084/jem.20070489 PMC2118691

[46] Yewdell JW, Bennink JR (1999) Immunodominance in major histocompatibility complex class I-restricted T lymphocyte responses. Annu Rev Immunol 17: 51–88. 10358753 10.1146/annurev.immunol.17.1.51

[47] Gomez CE, Najera JL, Perdiguero B, Garcia-Arriaza J, Sorzano CO, et al (2011) The HIV/AIDS vaccine candidate MVA-B administered as a single immunogen in humans triggers robust, polyfunctional, and selective effector memory T cell responses to HIV-1 antigens. J Virol 85: 11468–11478. 21865377 10.1128/JVI.05165-11 PMC3194965

[48] Tan AC, Eriksson EM, Kedzierska K, Deliyannis G, Valkenburg SA, et al (2012) Polyfunctional CD8(+) T cells are associated with the vaccination-induced control of a novel recombinant influenza virus expressing an HCV epitope. Antiviral Res 94: 168–178. 22504097 10.1016/j.antiviral.2012.03.009

[49] Rodriguez D, Gonzalez-Aseguinolaza G, Rodriguez JR, Vijayan A, Gherardi M, et al (2012) Vaccine efficacy against malaria by the combination of porcine parvovirus-like particles and vaccinia virus vectors expressing CS of Plasmodium. PLoS One 7: e34445. 22529915 10.1371/journal.pone.0034445 PMC3328484

[50] Elvang T, Christensen JP, Billeskov R, Thi Kim Thanh Hoang T, Holst P, et al (2009) CD4 and CD8 T cell responses to the M. tuberculosis Ag85B-TB10.4 promoted by adjuvanted subunit, adenovector or heterologous prime boost vaccination. PLoS One 4: e5139. 19357780 10.1371/journal.pone.0005139 PMC2663846

[51] Vasconcelos JR, Dominguez MR, Araujo AF, Ersching J, Tararam CA, et al (2012) Relevance of long-lived CD8(+) T effector memory cells for protective immunity elicited by heterologous prime-boost vaccination. Front Immunol 3: 358. 23264773 10.3389/fimmu.2012.00358 PMC3525016

[52] Michailowsky V, Silva NM, Rocha CD, Vieira LQ, Lannes-Vieira J, et al (2001) Pivotal role of interleukin-12 and interferon-gamma axis in controlling tissue parasitism and inflammation in the heart and central nervous system during Trypanosoma cruzi infection. Am J Pathol 159: 1723–1733. 11696433 10.1016/s0002-9440(10)63019-2 PMC3277321

[53] Rodrigues AA, Saosa JS, da Silva GK, Martins FA, da Silva AA, et al (2012) IFN-gamma plays a unique role in protection against low virulent Trypanosoma cruzi strain. PLoS Negl Trop Dis 6: e1598. 22509418 10.1371/journal.pntd.0001598 PMC3317909

[54] Takayama E, Ono T, Carnero E, Umemoto S, Yamaguchi Y, et al (2010) Quantitative and qualitative features of heterologous virus-vector-induced antigen-specific CD8+ T cells against Trypanosoma cruzi infection. Int J Parasitol 40: 1549–1561. 20620143 10.1016/j.ijpara.2010.05.011 PMC2952726

[55] Marinho CR, Nunez-Apaza LN, Martins-Santos R, Bastos KR, Bombeiro AL, et al (2007) IFN-gamma, but not nitric oxide or specific IgG, is essential for the in vivo control of low-virulence Sylvio X10/4 Trypanosoma cruzi parasites. Scand J Immunol 66: 297–308. 17635807 10.1111/j.1365-3083.2007.01958.x

[56] Dominguez MR, Ersching J, Lemos R, Machado AV, Bruna-Romero O, et al (2012) Re-circulation of lymphocytes mediated by sphingosine-1-phosphate receptor-1 contributes to resistance against experimental infection with the protozoan parasite Trypanosoma cruzi. Vaccine 30: 2882–2891. 22381075 10.1016/j.vaccine.2012.02.037

[57] Silverio JC, Pereira IR, Cipitelli Mda C, Vinagre NF, Rodrigues MM, et al (2012) CD8+ T-cells expressing interferon gamma or perforin play antagonistic roles in heart injury in experimental Trypanosoma cruzi-elicited cardiomyopathy. PLoS Pathog 8: e1002645. 22532799 10.1371/journal.ppat.1002645 PMC3330123

[58] Silverio JC, de-Oliveira-Pinto LM, da Silva AA, de Oliveira GM, Lannes-Vieira J (2009) Perforin-expressing cytotoxic cells contribute to chronic cardiomyopathy in Trypanosoma cruzi infection. Int J Exp Pathol 91: 72–86. 19878357 10.1111/j.1365-2613.2009.00670.x PMC2812730

[59] Vasconcelos JR, Bruna-Romero O, Araujo AF, Dominguez MR, Ersching J, et al (2012) Pathogen-induced proapoptotic phenotype and high CD95 (Fas) expression accompany a suboptimal CD8+ T-cell response: reversal by adenoviral vaccine. PLoS Pathog 8: e1002699. 22615561 10.1371/journal.ppat.1002699 PMC3355083

[60] Hamada H, Bassity E, Flies A, Strutt TM, Garcia-Hernandez Mde L, et al (2013) Multiple redundant effector mechanisms of CD8+ T cells protect against influenza infection. J Immunol 190: 296–306. 23197262 10.4049/jimmunol.1200571 PMC3864858

