# Supplementary figures and images for: Vaccination Using Recombinants Influenza and Adenoviruses Encoding Amastigote Surface Protein-2 Are Highly Effective on Protection against Trypanosoma cruzi Infection
Source: PLoS One. 2013 Apr 24;8(4):e61795. doi: 10.1371/journal.pone.0061795 (PMC3634828; doi:10.1371/journal.pone.0061795)

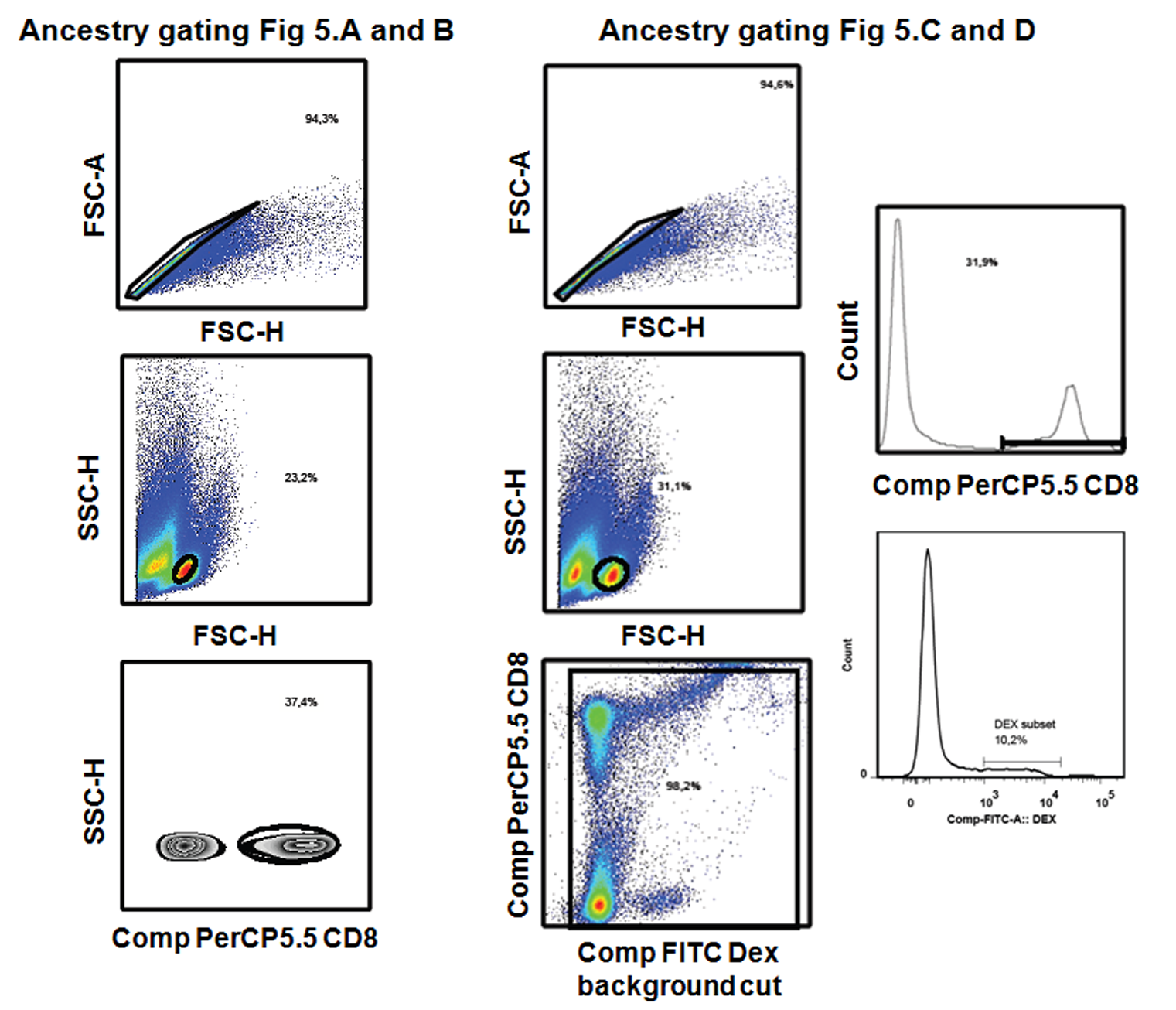

Supplement: Figure S1 — Representative of ancestry gates for flow cytometry experiments. Correspondent ancestry gates for the figure 5 analysis. (TIF) [file pone.0061795.s001.tif]

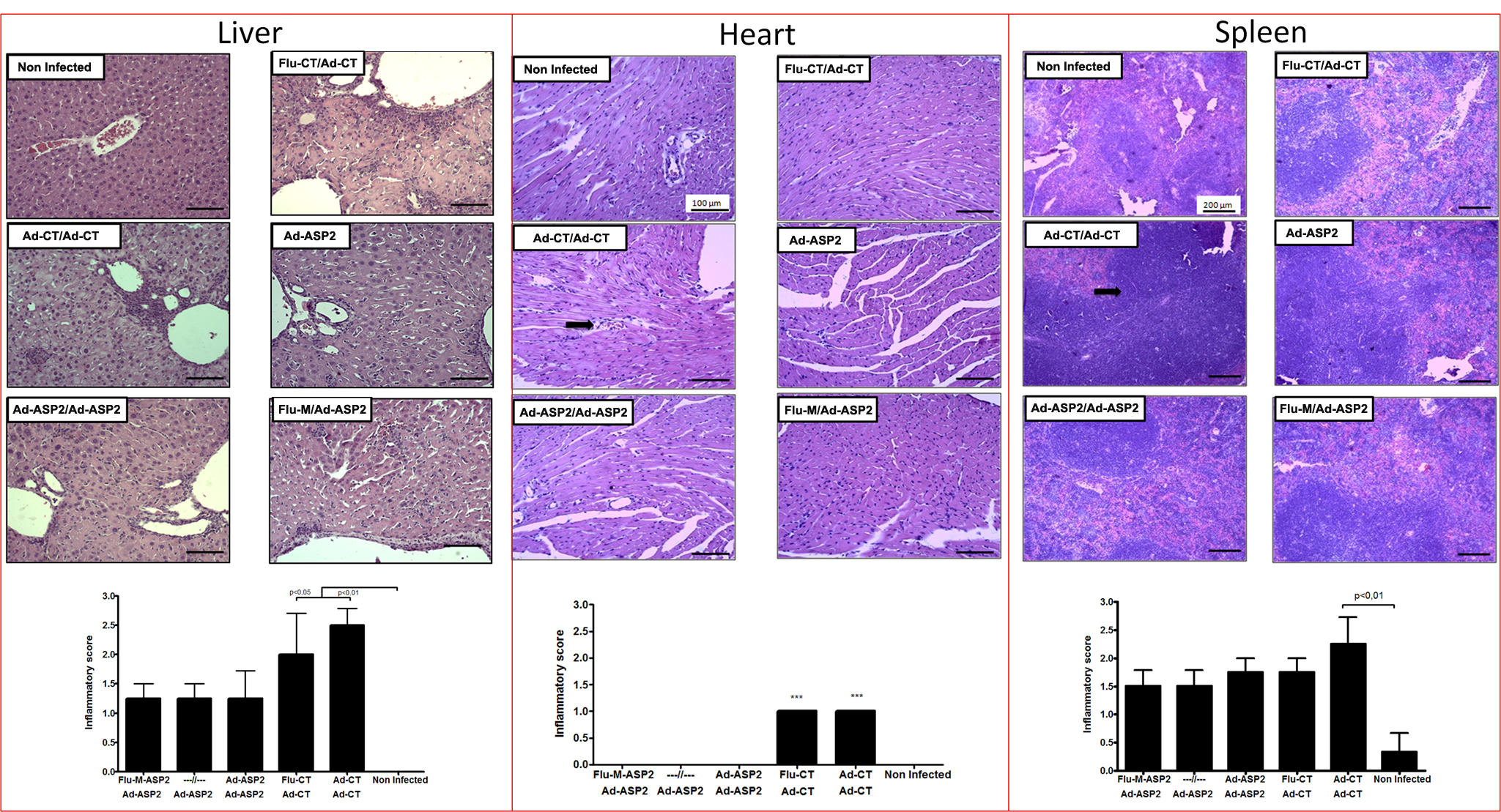

Supplement: Figure S2 — Histopathological analyses of liver, spleen and heart derived from infected mice. Male C3H/He mice were primed and boosted according different immunization protocols and infected with 500 bloodstream trypomastigotes of Y strain of T. cruzi. Fifteen days after the infection, mice were euthanized and spleen, liver and heart were harvested, fixed and processed for histopathology. The organ sections were stained using hematoxilin-eosin and the degree of tissue inflammation was evaluated (scale bar - 100 µm). (TIF) [file pone.0061795.s002.tif]

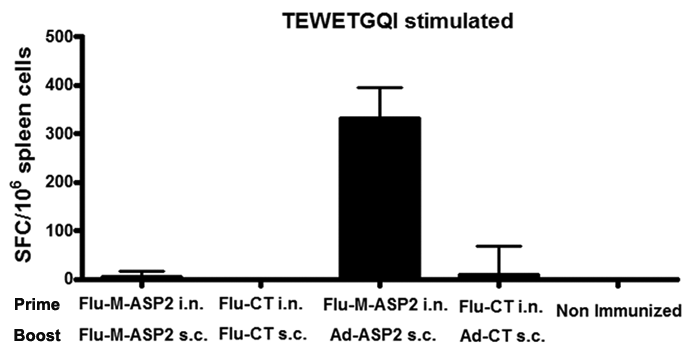

Supplement: Figure S3 — Cellular responses to immunodominant epitope from ASP2 in mice immunized twice using Flu-M-ASP2. ELISPOT of stimulated splenocytes taken from C3H/He mice immunized with the depicted protocols. The prime-boost was performed within an interval of 28 days and the experiment was performed 21 days post boost. The splenocytes were incubated 18 h in the presence of 10 µg of TEWETGQI peptide (n = 5 for all groups except non-immunized group NI/NI, n = 3). (TIF) [file pone.0061795.s003.tif]

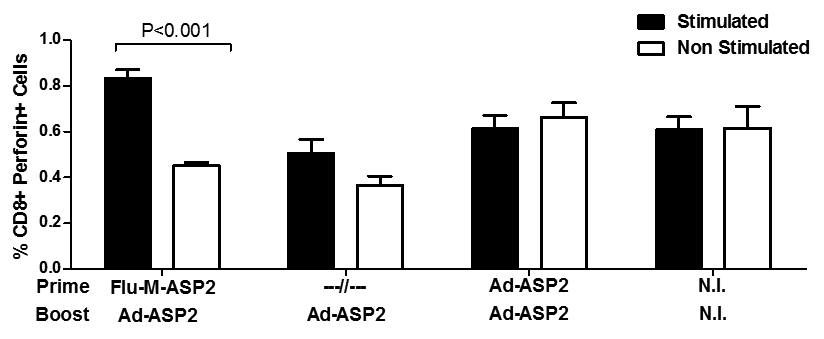

Supplement: Figure S4 — Perforin production in splenocytes derived from C3H/He immunized mice. Splenocytes derived from immunized C3H/He mice were ex vivo stimulated or not in the presence of Brefeldin A and Monesin A and the immunodominant peptide TEWETGQI for 12 hours, prepared, labeled and submitted to flow cytometry (n = 4). N.I. Non-immunized/Non-infected. Their staining profiles were analyzed using FlowJo and statistical analysis performed was 2-Way ANOVA with Bonferroni post-test using GraphPad Prism 5.0 Software. (TIF) [file pone.0061795.s004.tif]
